# Financial incentives for human resources for health: What do we know? What do we do? The case of Sierra Leone

**DOI:** 10.1186/1472-6963-14-S2-O22

**Published:** 2014-07-07

**Authors:** Maria Paola Bertone

**Affiliations:** 1 ReBUILD Consortium & Department of Global Health and Development, London School of Hygiene and Tropical Medicine, London, UK

## Background

Human resources for health (HRH) represents an essential component for well-functioning health systems. In recent years, there has been increased attention on the determinants of health workers (HWs) motivation, and in particular the role of financial incentives. Some countries have embarked on reforms to increase salaries and revise incentive packages, for example introducing rural allowance and/or performance-based financing. Sierra Leone provides an interesting case as, since 2009, a series of HRH reforms have been introduced, linked with the Free Healthcare Initiative.

## Materials and methods

Twenty-three key informant interviews at central level and 18 interviews at district level in Bo, Kenema and Moyamba were carried out, with staff from the Ministry of Health and Sanitation, development partners and local and international NGOs. Additionally, a survey of 266 HWs and in-depth interviews with 32 HWs were carried out in the same three districts. Making use of these data and policy analyses tools, the study looks in turn at (i) decision-making at central level, (ii) implementation of HRH policies in the districts, and (iii) impact on individual HWs incentives.

## Results

The case study illustrates the challenges in the process of transformation of evidence into national policies, and policies into effective practices at local level. In the case of Sierra Leone, the initial evidence-base upon which HRH reforms were introduced was relatively thin. However, these changes are generally recognized, especially by actors at central level, as ‘successful’ in addressing some of the HRH challenges as well as contributing to the Free Healthcare Initiative. At district level, other factors re-shaped the HRH strategies. In particular, the number, type and focus of external organizations present in the districts created different ‘political economy’ dynamics and contexts for the implementation of the reforms, which influenced their functioning and effects. Finally, the HRH measures eventually interacted with the existing district-specific incentive environment for HWs, affecting their performance in different ways.

## Conclusions

The analysis allows reflecting on the role and the ‘political space’ available for evidence-based policy-making in post-conflict, data-poor environments, and in particular in situations where there is tension between the role of evidence and the essentially political concerns of a brief, fast-moving ‘window of opportunity’ for reform. Moreover, moving to the local level, the study highlights how the local context and the presence of different actors and pre-existing incentives affect the actual functioning of the measures and influence their impact on HWs behaviour.

